# Lipid Metabolism Disorders as Diagnostic Biosignatures in Sepsis

**DOI:** 10.3390/idr16050062

**Published:** 2024-08-26

**Authors:** Charlotte Birner, Patricia Mester, Gerhard Liebisch, Marcus Höring, Stephan Schmid, Martina Müller, Vlad Pavel, Christa Buechler

**Affiliations:** 1 Department of Internal Medicine I, Gastroenterology, Hepatology, Endocrinology, Rheumatology, and Infectious Diseases, University Hospital Regensburg, 93053 Regensburg, Germany; charlotte.birner@stud.uni-regensburg.de (C.B.); patricia.mester@klinik.uni-regensburg.de (P.M.); stephan.schmid@klinik.uni-regensburg.de (S.S.); martina.mueller-schilling@klinik.uni-regensburg.de (M.M.); vlad.pavel@klinik.uni-regensburg.de (V.P.); 2 Institute of Clinical Chemistry and Laboratory Medicine, Regensburg University Hospital, 93053 Regensburg, Germany; gerhard.liebisch@klinik.uni-regensburg.de (G.L.); marcus.hoering@klinik.uni-regensburg.de (M.H.)

**Keywords:** apolipoprotein, triglyceride, cholesterol, septic shock, survival

## Abstract

Critical illness causes disturbances in lipid metabolism. Here, we investigated the levels of apolipoprotein A-IV (apoA-IV), a regulator of triglyceride and cholesterol metabolism, in human sepsis. ApoA-IV (analyzed in 156 patients with systemic inflammatory response syndrome (SIRS)/sepsis) and cholesteryl ester (CE) (analyzed in 121 of these patients) were lower in patients compared to 43 healthy controls. In contrast, triglyceride (TG) levels were elevated in patients. ApoA-IV levels in plasma of the patients did not correlate with these lipids. Patients with SIRS, sepsis or septic shock had comparable apoA-IV, TG, CE and free cholesterol (FC) levels. Patients on dialysis had significantly lower CE levels, whereas apoA-IV levels did not change much. CE levels were elevated in patients with viral sepsis due to SARS-CoV-2 infection in comparison to SIRS/sepsis patients not infected by this virus. CE levels correlated negatively with procalcitonin, interleukin-6 and bilirubin, while TGs were positively associated with bilirubin and C-reactive protein. ApoA-IV, TG, CE and FC levels were not associated with bacterial infection or survival. In conclusion, this analysis suggests that CE levels decline in sepsis-related renal failure and also shows that plasma apoA-IV and CE levels are early biomarkers of sepsis.

## 1. Introduction

Sepsis is associated with several alterations in lipid metabolism, including a decrease in low-density lipoprotein (LDL) and high-density lipoprotein (HDL) cholesterol levels. Apolipoprotein (apo) A-I is the most abundant and apoA-II is the second most abundant apolipoprotein in HDL and apoB-100 is the major apolipoprotein of LDL [1,2]. Apolipoprotein levels also change during sepsis, and apolipoprotein (apo) A-I and apo A-II are reduced. A proteomic analysis observed lower plasma apoB-100 levels in patients with sepsis compared to healthy controls [3,4,5,6,7,8].

In addition to cholesterol, triglycerides (TGs) are essential components of lipoproteins [1,2]. Inflammatory cytokines increase hepatic TG synthesis and suppress fatty acid oxidation and TG clearance, and accordingly, fatty acid and TG levels are increased in sepsis. In addition, lipolysis in peripheral tissues is stimulated during sepsis, further increasing blood fatty acid levels [9].

Infection with severe acute respiratory syndrome coronavirus type 2 (SARS-CoV-2) can cause viral sepsis [10,11]. Patients with COVID-19 had lower levels of total cholesterol, LDL cholesterol, HDL cholesterol, apoA-I, apoA-II, apoA-IV and apoB [12,13]. Blood TG levels were either elevated or normal. With recovery from the disease, lipoproteins returned to pre-infection levels [13]. However, the causes of lipid and lipoprotein abnormalities in patients with severe illness and their relevance are not well understood [3,4,5,6,7,8].

Apolipoprotein A-IV (apoA-IV) is mainly synthesized in the small intestine and binds lipids [14]. Upon ingestion of long-chain fatty acids, apoA-IV is packaged into nascent chylomicrons in intestinal enterocytes and secreted into the intestinal lymph. During metabolism of chylomicrons in the blood, about 25% of apoA-IV is transferred to HDL and the remainder is found in a lipid-free form [14,15]. Despite in vitro evidence for an essential role of apoA-IV in fatty acid uptake, the majority of in vivo studies have demonstrated that apoA-IV is not required for lipid absorption [15]. Plasma apoA-IV levels exhibit a moderate increase following fat ingestion, reaching 114 to 117% of the fasting value and peaking at 4 to 5 h after the meal [16,17]. ApoA-IV can promote efflux of cellular cholesterol and uptake of cholesterol by the liver, thus contributing to reverse cholesterol transport, a pathway that protects from atherosclerosis [14,15].

ApoA-IV also exerts anti-oxidative and anti-inflammatory functions [14,15]. The expression of human apoA-IV in apoE-deficient mice resulted in a reduced production of inflammatory factors upon lipopolysaccharide challenge [18]. Furthermore, the daily injection of recombinant human apoA-IV also delayed the onset and diminished the inflammatory response in experimental colitis. The greater susceptibility of apoA-IV null mice to colitis was found to be reversed by the administration of exogenous apoA-IV [19]. The protective effects of apoA-IV in a rabbit model for vascular inflammation were attributed to the inhibition of the translocation of the p65 subunit of nuclear factor-κB to the cell nucleus [20]. ApoA-IV was also found to prevent allergen-driven airway inflammation in a mouse model. Accordingly, apoA-IV serum levels were found to be significantly lower in patients with allergies in comparison to controls [21].

A proteomic analysis observed reduced plasma apoA-IV levels in patients with pneumogenic sepsis in comparison to healthy controls [8]. However, mice challenged with lipopolysaccharide displayed increased apoA-IV in HDL and higher apoA-IV expression in the liver in comparison to control animals [22]. Serum levels of apoA-IV were increased in patients with mild human adenovirus community-acquired pneumonia compared to healthy controls and were further induced in severe cases, showing an association with disease severity [23]. The relationship between blood apoA-IV levels and severe disease is unclear, according to the results of the current studies.

Kidney injury is a common complication of sepsis [24]. ApoA-IV has been identified as an early marker of kidney impairment in the general population and patients with primary chronic kidney disease [25,26]. In patients with chronic kidney diseases, inflammatory cytokine and apoA-IV levels in serum were higher in comparison to healthy controls [27]. Diabetic nephropathy was also associated with a nearly two-fold increase of serum apoA-IV [28]. To our knowledge, the analysis of plasma apoA-IV levels in septic patients and their associations with sepsis-induced kidney failure has not yet been explored. This study aims to address this research gap. Furthermore, our study aimed to determine the associations between plasma apoA-IV levels, sepsis-associated dyslipidaemia, causes of severe illness, severity of illness and survival in a large cohort of patients with systemic inflammatory response syndrome (SIRS)/sepsis.

## 2. Materials and Methods

### 2.1. Patients

Between August 2018 and January 2024, plasma was collected from 156 patients admitted to the intensive care unit at the University Hospital of Regensburg. According to the Sepsis-3 criteria, 39 patients were diagnosed with sepsis, while 78 were diagnosed with septic shock [29]. The remaining 39 patients did not meet the Sepsis-3 criteria and were diagnosed with systemic inflammatory response syndrome (SIRS) [30]. These patients had a sepsis-related organ failure assessment score below 2 based on the Sepsis-3 criteria and thus did not meet the criteria for sepsis [29,30]. Patients with multi-resistant infections and patients with viral hepatitis or human immunodeficiency virus infection were excluded.

Common comorbidities were neoplasms such as colorectal cancer, cholangiocellular carcinoma and adenocarcinoma (13.5%), autoimmune diseases such as Hashimoto’s thyroiditis and Sjögren’s syndrome (7.7%), haematological diseases such as acute promyelocytic leukaemia and acute lymphoblastic leukaemia (7.7%). A total of 7.1% of patients were immunosuppressed after organ transplantation. The control group comprised 21 healthy females and 22 healthy males, with more females compared to the patient group (*p* = 0.017). The age range of the control group was 56 (21–86) years, and the healthy controls were younger compared to the patient group (*p* = 0.031).

### 2.2. Analysis of ApoA-IV

Blood samples were taken from patients within 12 to 24 h of admission to the intensive care unit. EDTA was used as the anticoagulant and plasma was separated from the blood samples. Aliquots were stored at −80 °C and were thawed immediately prior to utilization. The apoA-IV ELISA was obtained from Antibodies Online (catalogue number: ABIN705996, Aachen, Germany) and was performed exactly according to the protocol provided and recommended by the distributor, which is available online. Plasma was diluted 1:2-fold for analysis. All samples and standards were measured in duplicate and the mean values were used for calculations.

Information on the apoA-IV ELISA is provided by the company. Intra-assay precision was determined by replicating 3 samples with low, medium and high levels of apoA-IV 20 times on one plate. The coefficient of variation was <10%. Inter-assay precision was tested using 3 samples with low, medium and high levels of apoA-IV on 3 different plates with 8 replicates in each plate. The coefficient of variation was <12%. The company also provides immunoblot validation data for the kit components.

### 2.3. Analysis of Cholesterol and TG

For quantitative lipidomics, non-natural standards were added to the plasma samples. Ten µL of plasma was used for lipid extraction following the protocol of Bligh and Dyer [31].

The analysis of triglycerides (TGs), cholesteryl ester (CE) and free cholesterol (FC) was operated by flow injection analysis Fourier-transform mass spectrometry (FIA-FTMS) on a high-resolution hybrid quadrupole-Orbitrap mass spectrometer [32]. A comprehensive protocol of the FIA-FTMS method was recently published [33]. In short, TG and CE were analyzed in positive ion mode *m/z* 500–1000. CE were corrected for their species-specific responses [34]. A multiplexed acquisition approach was employed to analyze FC and the internal standard (FC[D7]) simultaneously [34].

TG, CE and FC levels in plasma of 7 male and 10 female controls were also analyzed. In the control group, there were more females (*p* = 0.026), and the controls were younger (54 (28–78) years; *p* = 0.040) in comparison to our patients.

### 2.4. Statistical Analysis

Data in the figures are visualized as boxplots, with the minimum, maximum and median values, and the first and third quartiles. Outliers are indicated by circles (apoA-IV levels >1.5× the interquartile range from above the third quartile or below the first quartile) and asterisks (apoA-IV levels >3.0× the interquartile range from either quartile). Table data are the median values, minimum and maximum values. The statistical analyses were conducted using the Chi-Square test, Mann–Whitney U test, Kruskal–Wallis test and Spearman’s correlation (IBM SPSS Statistics 26.0). A *p*-value of less than 0.05 was deemed significant.

## 3. Results

### 3.1. Plasma ApoA-IV, Triglyceride and Cholesterol Levels of Controls and SIRS/Sepsis Patients

Table 1 summarizes the characteristics of the cohort of 156 patients with SIRS/sepsis in whom plasma apoA-IV was measured. The median age of the patients was 59 years and approximately one third were female (Table 1). The control group consisted of 43 healthy subjects, more of whom were female (*p* = 0.017) and younger than the patient group (*p* = 0.031).

**Figure 1:**
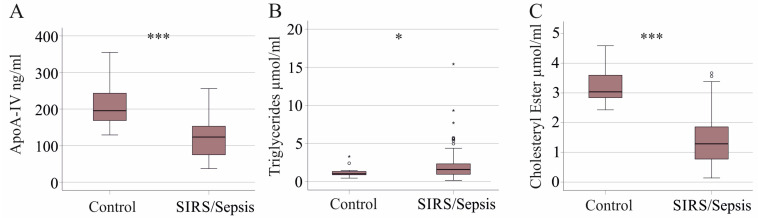
Plasma apoA-IV and lipid levels of healthy controls and SIRS/sepsis patients. (**A**) Plasma apoA-IV levels of controls and SIRS/sepsis patients; (**B**) plasma triglyceride levels of SIRS/sepsis patients with liver cirrhosis patients excluded; (**C**) plasma cholesteryl ester levels of SIRS/sepsis patients with liver cirrhosis patients excluded. * *p* < 0.05, *** *p* < 0.001.

The plasma apoA-IV levels in the 156 SIRS/sepsis patients were 140 (54–273) ng/mL and were lower than in the 43 controls with 195 (129–355) ng/mL (Figure 1A). Patients without and with liver cirrhosis had similar apoA-IV levels (*p* = 0.292).

In the plasma of 150 of the 156 SIRS/sepsis patients, cholesteryl ester (CE), free cholesterol (FC) and TG were measured. The plasma FC (*p* = 0.255) and TG levels of patients and controls were similar (*p* = 0.119), and CE levels of patients were low (*p* < 0.001). SIRS/sepsis patients with liver cirrhosis (29 patients) had reduced triglyceride (*p* < 0.001), FC (*p* = 0.039) and CE (*p* < 0.001) levels in comparison to SIRS/sepsis patients with normal liver function. For this reason, patients with liver cirrhosis were excluded from analysis regarding lipids. The details of this subcohort are also given in Table 1. In the cohort without liver cirrhosis, plasma TG levels of SIRS/sepsis patients were higher (*p* = 0.017) and plasma CEs were reduced (*p* < 0.001) in comparison to controls (Figure 1B,C). FC levels of patients and controls were almost identical (*p* = 0.556).

Serum apoA-IV levels were not correlated with TG (r = −0.151, *p* = 0.100), CE (r = −0.070, *p* = 0.449) and FC levels (r = −0.072, *p* = 0.432).

In the entire cohort, apoA-IV did not correlate with alanine aminotransferase (ALT), aspartate aminotransferase (AST), albumin or bilirubin and negatively correlated with gamma glutamyltransferase (gamma GT) (r = −0.200, *p* = 0.024).

In the cohort excluding patients with liver cirrhosis CE levels correlated with bilirubin (r = −0.428, *p* < 0.001), albumin (r = 0.233, *p* = 0.014), AST (r = −0.204, *p* = 0.034) and gamma GT (r = 0.210, *p* = 0.039). FC (r = 0.343, *p* < 0.001) and TG levels (r = 0.216, *p* = 0.022) correlated with bilirubin. FC also correlated with AST (r = 0.213, *p* = 0.026), ALT (r = 0.227, *p* = 0.019) and gamma GT (r = 0.296, *p* = 0.003).

### 3.2. Plasma ApoA-IV and Lipid Levels of SIRS/Sepsis Patients in Relation to Sex, Age and BMI

No significant sex differences in plasma apoA-IV levels (*p* = 0.282), TG (*p* = 0.519), CE (*p* = 0.461) and FC (*p* = 0.858) levels were found in the patient group. There was no correlation between age and apoA-IV levels among patients, with a Spearman’s correlation coefficient of r = −0.083 (*p* = 0.243). TG but not CE and FC negatively correlated with age (r = −0.273, *p* = 0.002). Plasma ApoA-IV and lipids did not correlate with BMI (*p* > 0.05 for all).

### 3.3. Plasma ApoA-IV and Lipid Levels of SIRS/Sepsis Patients in Relation to SIRS, Sepsis and Septic Shock

**Figure 2:**
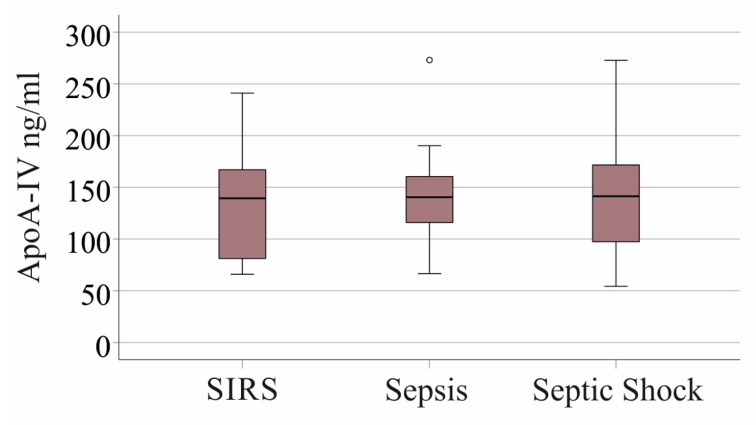
ApoA-IV in plasma of patients with SIRS, patients with sepsis and patients with septic shock.

The plasma apoA-IV levels of patients with SIRS, sepsis and septic shock were constant (Figure 2). TG (*p* = 0.931), CE (*p* = 0.429) and FC (*p* = 0.739) levels were also similar between these three groups. These findings demonstrate that disorders in lipid metabolism do not appear late when a septic state is established but already in the early stages of the disease that trigger sepsis.

### 3.4. Plasma ApoA-IV and Lipid Levels of SIRS/Sepsis Patients in Respect to Preexisting Diseases and SARS-CoV-2 Infection

**Figure 3:**
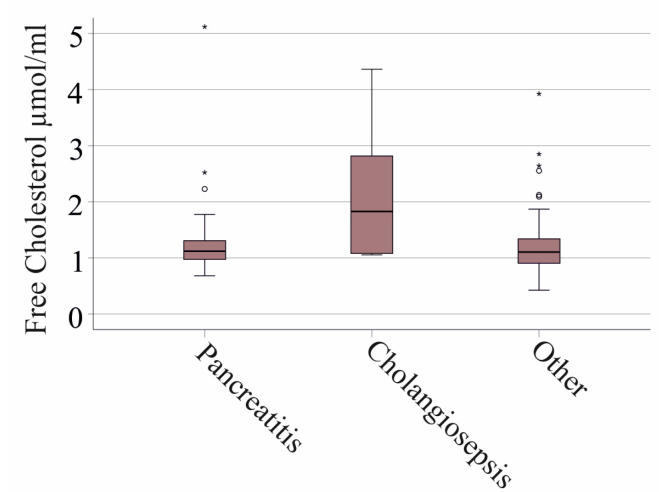
Free cholesterol levels in plasma of patients with pancreatitis or cholangitis and patients without these underlying diseases (other).

Underlying conditions may change the abundance of biomarkers in sepsis. Thirty-five of our patients had pancreatitis and 13 cholangitis. Plasma apoA-IV levels showed no difference between these groups (*p* = 0.722). TG (*p* = 0.296) and CE levels (*p* = 0.867) of patients with pancreatitis (30 patients), cholangitis (nine patients) and patients without these diseases were also similar. FC levels tended to be increased in cholangiosepsis (*p* = 0.051, Figure 3).

The 40 patients with pulmonary infections and the 13 patients with urosepsis had comparable lipid levels (*p* > 0.05 for all). ApoA-IV levels of the 51 patients with pulmonary infections and the 14 patients with urosepsis were comparable (*p* = 0.218).

**Figure 4:**
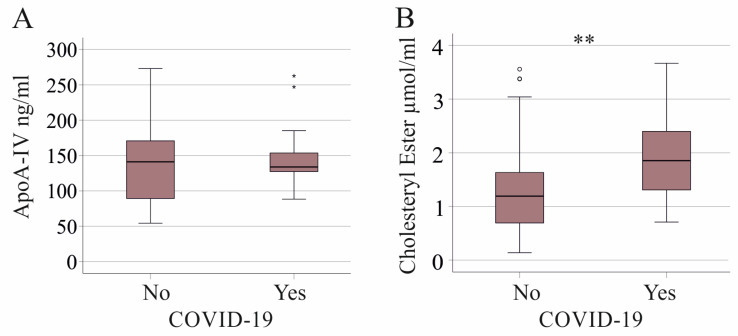
ApoA-IV and cholesteryl ester levels in plasma of critically ill patients without (No) and with (Yes) SARS-CoV-2 infection. (**A**) Plasma apoA-IV levels; (**B**) cholesteryl ester levels. ** *p* < 0.01.

Twenty-three patients had SARS-CoV-2 infection which progressed to septic shock and acute respiratory distress syndrome. Plasma apoA-IV levels showed no difference between these groups (*p* = 0.504, Figure 4A). The 21 COVID-19 patients in the cohort where lipids have been measured in plasma had higher CE levels compared with SIRS/sepsis patients without SARS-CoV-2 infection (*p* = 0.004; Figure 4B). It should be noted that patients with COVID-19 sepsis still had lower CE levels in comparison to healthy controls (*p* < 0.001), whereas TG levels were induced (*p* = 0.026).

FC (*p* = 0.898) and TG (*p* = 0.816) levels did not differ between SIRS/sepsis patients without and with SARS-CoV-2 infection. COVID-19 patients had lower CRP (*p* = 0.046) and procalcitonin (*p* = 0.018) levels compared to SIRS/sepsis patients without this viral infection. Patients with COVID-19 also had higher albumin (*p* < 0.001) and lower serum bilirubin (*p* < 0.001) levels, while aminotransferase levels did not differ between these groups.

### 3.5. Plasma ApoA-IV and Lipid Levels in Respect to Vasopressor Therapy and Interventions

**Figure 5:**
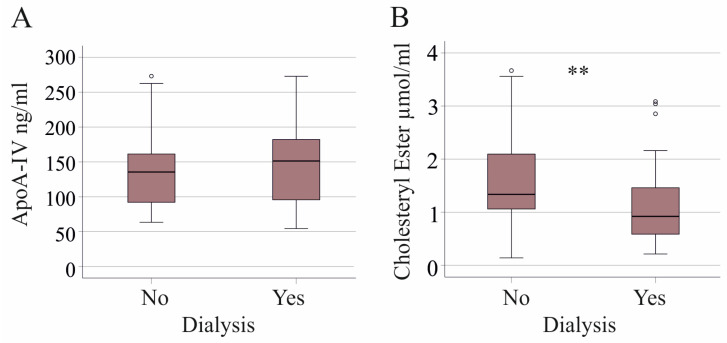
ApoA-IV and cholesteryl ester levels in plasma of SIRS/sepsis patients without (No) and with (Yes) dialysis. (**A**) Plasma apoA-IV levels; (**B**) cholesteryl ester levels. ** *p* < 0.01.

Plasma apoA-IV, TG, CE and FC levels in our SIRS/sepsis cohort were similar between the patients treated with mechanical ventilation and those treated with vasopressor therapy (Table 2). Patients on dialysis tended to have higher apoA-IV (*p* = 0.082) and had significantly reduced CE levels (Table 2 and Figure 5). FC levels (*p* = 0.281) did not change with dialysis.

### 3.6. Plasma ApoA-IV, Lipids and Inflammation Markers

In our SIRS/sepsis cohort plasma, apoA-IV was not correlated with leukocytes, CRP, procalcitonin or IL-6. TGs positively correlated with lymphocyte count, immature granulocytes and CRP. CE levels were negatively associated with monocyte count, procalcitonin and IL-6. FC positively correlated with CRP (Table 3).

### 3.7. Plasma ApoA-IV and Lipids in Bacterial Infections

In SIRS/sepsis patients, plasma apoA-IV levels among patients without detectable bacteria in the blood, those in the patients infected with Gram-negative bacteria (24 patients), those in the patients with Gram-positive bacteria (24 patients) and of patients co-infected with both types of bacteria (four patients) were similar (*p* = 0.804). TG (*p* = 0.300), CE (*p* = 0.206) and FC (*p* = 0.082) levels were not changed with bacterial infection.

### 3.8. Plasma ApoA-IV, Lipids and Survival

Plasma apoA-IV levels were similar in the 36 non-survivors compared to the survivors (*p* = 0.581).

**Figure 6:**
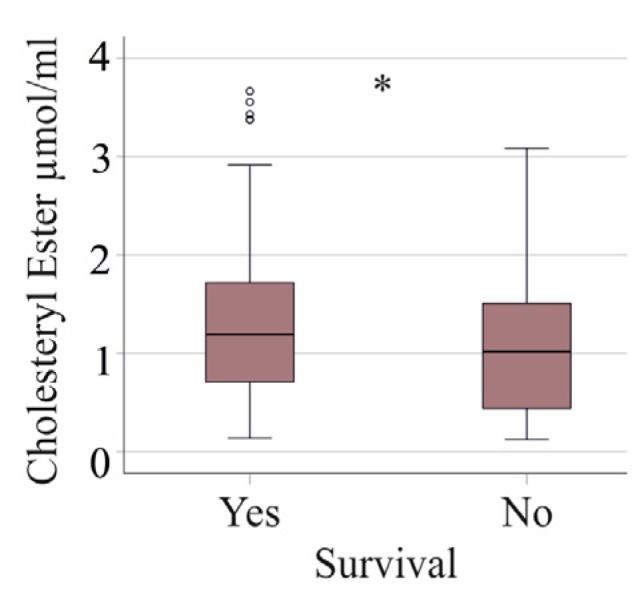
Cholesteryl ester levels in plasma of all critically ill patients, which means that patients with liver cirrhosis were included, in survivors (Yes) and non-survivors (No). * *p* < 0.05.

TG levels of the 25 non-survivors (*p* = 0.318), as well as CE (*p* = 0.243) and FC (*p* = 0.676) levels, were similar in comparison to survivors. Including patients with liver cirrhosis revealed reduced CE levels of non-survivors (*p* = 0.035, Figure 6).

## 4. Discussion

**Figure 7:**
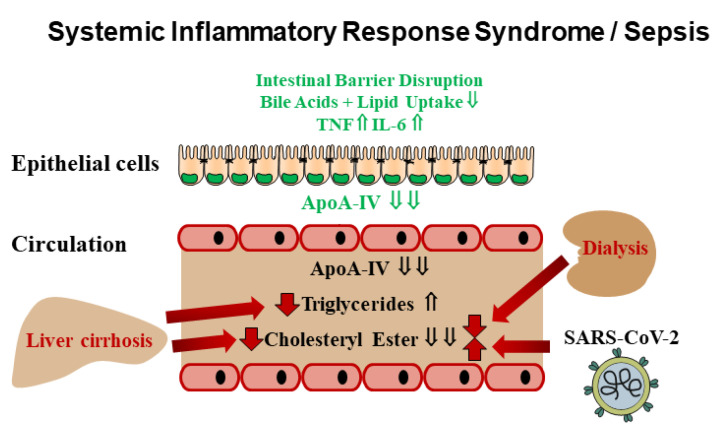
Summary of the study. Sepsis compromises the integrity of the epithelial barrier, thereby increasing intestinal permeability [37]. IL-6 and tumor necrosis factor have been described to reduce apoA-IV secretion from differentiated Caco2 cells [38]. Bile acid excretion is impaired in sepsis, which may reduce fatty acid uptake associated with apoA-IV induction [39]. The current analysis showed low apoA-IV and cholesteryl ester levels in patients with SIRS/sepsis compared to controls, whereas triglycerides are elevated. Dialysis is associated with lower plasma cholesteryl ester levels in critically ill patients, and cirrhosis is associated with lower triglyceride and cholesteryl ester levels. Sepsis patients with SARS-CoV-2 infection had higher cholesteryl ester levels compared to sepsis patients not infected with this virus. (⇓) reduced, (⇓ ⇓) strongly reduced, (⇑) increased.

Our analysis showed that SIRS/sepsis patients had reduced plasma apoA-IV levels compared with healthy controls. Consistent with previous reports, CE levels were low, and TGs were elevated [7,35,36] (Figure 7). Plasma apoA-IV did not correlate with these lipids, measures of inflammation or disease severity.

Of clinical importance, disorders in lipid metabolism occur both in established sepsis and in the early phases of diseases that may lead to sepsis. This finding is novel and has not been previously described. Thus, low plasma apoA-IV levels can indicate an infection in the early stages, even without clinical signs of sepsis, allowing for timely therapeutic interventions.

The primary site of apoA-IV synthesis is the small intestine (Figure 7), and apoA-IV is released into the lymph during lipid absorption [15]. Low plasma levels of apoA-IV in sepsis suggest that intestinal synthesis is impaired. Sepsis affects the integrity of the epithelial barrier, thereby increasing intestinal permeability [37]. Inflammation contributes to the disruption of the intestinal barrier in sepsis [37,40]. Patients with inflammatory bowel disease have greatly reduced apoA-IV protein in the ileum and colon [41]. IL-6 and tumor necrosis factor have been described to reduce apoA-IV secretion from differentiated Caco2 cells [38] (Figure 7). E-cadherin is essential for maintaining the intestinal barrier and is low in the inflamed intestine [37,40]. E-cadherin induces apoA-IV expression in intestinal epithelial cells [42]. Thus, it is likely that low apoA-IV in plasma of patients with sepsis is related to inflammation and intestinal barrier disruption. Excretion of bile acids is impaired in sepsis, which may reduce fatty acid uptake associated with an induction of apoA-IV [39] (Figure 7). Plasma apoA-IV negatively correlated with gamma GT, indicating that liver dysfunction may affect apoA-IV levels. However, this effect is modest because patients with and those without liver cirrhosis had similar plasma apoA-IV levels.

Liver cirrhosis is associated with hypolipoproteinemia, and both CE and TG levels were low in plasma of patients with SIRS/sepsis and liver cirrhosis [32,43] (Figure 7). How apoA-IV levels change in patients with cirrhosis is unclear. Wang et al. observed strongly increased serum apoA-IV levels in patients with early stages of liver fibrosis compared to healthy controls [44]. Seishima et al. reported decreased intestinal apoA-IV mRNA expression and normal serum levels in patients with liver cirrhosis [45]. In our SIRS/sepsis cohort, plasma apoA-IV levels were comparable in patients with and without cirrhosis, suggesting that cirrhosis does not have a major effect on the synthesis and/or clearance of this apolipoprotein, which also does not appear to contribute to the reduced lipid levels in cirrhosis.

In addition, plasma apoA-IV did not correlate with TG, CE and FC levels in SIRS/sepsis patients when patients with liver cirrhosis were excluded, also ruling out a role for apoA-IV in dyslipidemia in sepsis. Patients with cholangiosepsis had higher FC plasma levels compared to those with sepsis from other causes, though the difference was not statistically significant. This observation is clinically important and warrants further research.

Higher TG levels in sepsis were observed only after excluding patients with cirrhosis, who have low plasma lipid levels. In SIRS/sepsis patients without cirrhosis, TG levels and FC correlated positively with bilirubin. CE levels correlated positively with albumin and negatively with bilirubin. Thus, liver dysfunction seems to modulate plasma TG, CE and FC levels, and future studies of lipids in severe disease should take this into account. ApoA-IV has consistently been shown to protect against inflammation [19,20,21], suggesting that low levels in SIRS/sepsis contribute to higher levels of inflammatory markers. However, plasma apoA-IV levels did not correlate with CRP, procalcitonin and IL-6, and our analysis does not provide evidence for an anti-inflammatory effect of apoA-IV in sepsis.

Inflammation contributes to low cholesterol and elevated TGs in sepsis [4,36]. TGs were positively correlated with CRP, lymphocyte and immature granulocyte counts, and CE levels were negatively correlated with procalcitonin, IL-6 and monocyte counts. These associations reveal that plasma TG as well as CE levels are associated with inflammation. Notably, FC levels did not decrease in SIRS/sepsis compared to controls but were positively correlated with CRP.

The significant decrease in CE species in combination with unchanged FC levels may be caused by impaired lecithin-cholesterol acyltransferase (LCAT) activity, which converts FC into CEs [7]. LCAT activity was negatively correlated with CRP in sepsis [46], consistent with the positive correlation between FC and CRP.

In our study, COVID-19 patients had higher CE levels compared to sepsis patients without SARS-CoV-2 infection. The COVID-19 patients had lower levels of CRP and procalcitonin, and higher CE levels may, in part, reflect less inflammation in these patients. Moreover, patients with COVID-19 had lower bilirubin and higher serum albumin, indicating better liver function. Serum CE levels were positively correlated with albumin and negatively with bilirubin. Our study shows, for the first time, that plasma CE levels distinguish COVID-19 sepsis from other etiologies of sepsis. Future research should investigate if plasma CE is a reliable biomarker for viral infections in general.

COVID-19 patients in intensive care had lower apoA-IV levels compared to healthy controls [12]. This decrease appears due to severe illness rather than SARS-CoV-2 infection itself. In our cohort, plasma apoA-IV levels were similar in SIRS/sepsis patients with and without COVID-19. ApoA-IV, FC, CE and TG levels were not changed with bacterial infection. Patients with and without bacteria in their blood had similar levels of apoA-IV and of these lipids. This shows that disturbances of apoA-IV and these lipids are due to severe illness and are not a direct effect of bacterial infection.

In addition, apoA-IV levels in patients with sepsis were not associated with survival. This is consistent with the findings in patients with severe COVID-19 [12]. In our sepsis patients, plasma levels of CE, FC and TG were not associated with mortality. Various other studies have shown associations of low cholesterol levels with mortality in sepsis [35,47]. Liver cirrhosis was an exclusion criterion in four of the 24 trials included in a recent meta-analysis, while other studies excluded patients on lipid-lowering medication or patients with a history of dyslipidemia [47]. A decrease in serum CE levels in non-survivors was evident in our entire cohort, most likely due to the larger number of SIRS/sepsis patients included in this analysis.

Kidney disease is another condition that affects serum cholesterol levels [48]. Patients on hemodialysis in our cohort had low CE levels and a high mortality of 45%. A decrease in serum cholesterol in hemodialysis patients compared to the normal population has been reported in other studies [48,49]. Acute kidney injury is common in sepsis patients, with an incidence of about 50%, and severe disease is associated with a high risk of death [50]. The present analysis shows that lower CE levels could be a valuable biomarker for acute renal failure in sepsis. Monitoring of CE plasma levels, thus, could serve as an early biomarker to identify septic patients at risk of developing renal dysfunction or renal failure.

The association between low blood cholesterol levels and mortality in sepsis is, therefore, confounded by the inclusion of patients with liver cirrhosis and patients with acute kidney injury. Further studies that can address all underlying diseases, drugs and interventions targeting lipid metabolism will need to demonstrate the relationship between cholesterol levels and survival in sepsis.

ApoA-IV was positively correlated with creatinine in patients with severe COVID-19 disease [12]. Higher levels of apoA-IV were associated with poor renal function in population-based cohorts [51]. In our sepsis cohort, plasma apoA-IV levels of patients requiring dialysis were not significantly elevated, showing that kidney dysfunction in critical illness is not associated with apoA-IV levels.

When analyzing lipid metabolites and apolipoproteins, it is important to identify potential confounders such as sex or age. In our sepsis cohort, CE, FC, TG and apoA-IV did not differ between men and women and were not correlated with BMI. TG levels were even negatively correlated with age.

In the normal adult population, men have higher levels of LDL cholesterol and TG than women, but this changes after menopause, when LDL cholesterol increases in women [52]. TG levels increase with age in both sexes [52]. LDL-cholesterol and TGs are not correlated with BMI in the normal population [53,54]. In addition, apoA-IV was increased in obesity [55] and was also found to be inversely correlated with BMI in females [56]. Plasma apoA-IV positively correlated with age in both sexes [56]. This study included 723 participants [56], and our cohort may have been too small to identify such associations. Another explanation is that apoA-IV, CE, and FC levels in SIRS/sepsis patients are not linked to sex, age or BMI.

Our study has limitations. The subcohort of patients with COVID-19 was small, and this will limit the statistical power. Plasma was mostly not collected in a fasted state and only early after admission of the patients to the intensive care unit. Therefore, this study cannot provide data on apoA-IV and lipid levels during the course of SIRS/sepsis. BMI and laboratory values of controls were not documented. In addition, this is a single-center study, and the results may not apply to different regions.

## 5. Conclusions

Our analysis highlights lipid metabolism in SIRS and sepsis of various etiologies. We propose new biomarkers and lipid signatures for diagnosing critical illness. We confirmed low plasma apoA-IV in sepsis [8], suggesting its potential as a biomarker. Of clinical importance, disturbances in apoA-IV occur in early disease stages that may precede sepsis. Early identification of patients at risk of sepsis by novel biomarkers can thus trigger timely clinical interventions. CE plasma levels are low in sepsis but higher in COVID-19 sepsis, distinguishing COVID-19 sepsis from other aetiologies of sepsis. Future research should address if plasma CE can detect viral sepsis in general. Furthermore, this study is the first to suggest that plasma CE may detect acute septic renal failure as an early biomarker. Thus, CE levels show potential for diagnosing renal dysfunction and renal failure in critical illness.

## Figures and Tables

**Table 1:** Characteristics of the entire cohort, and of the subgroup of patients where plasma lipids have been measured. This latter cohort excluded patients with liver cirrhosis. IL-6 and laboratory measures of liver function were not recorded for all patients and the number of patients is indicated by the superscripted numbers.

Parameters	Whole Cohort	Subcohort (Patients with Liver Cirrhosis Excluded)
Males/Females	111/45	85/36
Age (years)	59 (21–93)	59 (21–93)
BMI (kg/m^2^)	27 (15–56)	26 (15–56)
C-reactive protein mg/L	163 (4–697)	183 (23–697)
Procalcitonin ng/mL	1.17 (0.05–270.00)	1.12 (0.05–270.00)
SIRS/Sepsis/Septic Shock	39/39/78	26/31/64
IL-6 pg/mL	89 (0–5702) ^148^	75 (0–5702) ^117^
Leukocytes n × 10^9^/L	10.31 (0.06–1586.00)	10.24 (0.06–246.94)
Neutrophils n/nL	7.68 (0.01–70.20)	7.34 (0–70.20)
Basophils n/nL	0.04 (0–0.90)	0.04 (0–0.90)
Eosinophils n/nL	0.10 (0–8.80)	0.13 (0–8.80)
Monocytes n/nL	0.75 (0–45.00)	0.73 (0–4500)
Lymphocytes n/nL	0.95 (0.08 –28.60)	1.07 (0.08–28.60)
Immature granulocytes n/nL	0.12 (0–6.19)	0.13 (0–6.19)
Aspartate aminotransferase U/L	46 (6–1703) ^143^	40 (6–1597) ^113^
Alanine aminotransferase U/L	32 (5–770) ^141^	32 (6–770) ^111^
Albumin g/L	23.0 (6.3–42.0) ^146^	23.0 (6.3–42.0) ^116^
Gamma glutamyltransferase U/L	124 (11–1266) ^127^	122 (11–1266) ^100^
Bilirubin mg/dL	0.30 (0.10–7.80) ^146^	0.30 (0.10–4.60) ^102^

**Table 2:** Plasma apoA-IV, triglyceride, cholesteryl ester (CE) and free cholesterol (FC) levels of patients on dialysis, ventilation or vasopressor therapy and of patients without these therapies. The number of patients is given in the first column in parenthesis; the first number is related to apoA-IV analysis and the second one to lipid analysis.

Intervention/Drug	ApoA-IV (ng/mL)		TG (µmol/mL)		CE (µmol/mL)		FC (µmol/mL)	
	No	Yes	No	Yes	No	Yes	No	Yes
Dialysis (54/38)	136 (63–273)	151 (54–273)	1.7 (0.2–15.4)	1.5 (0.3–9.3)	1.4 (0.1–3.7)	0.9 (0.2–3.1) *p* = 0.002	1.2 (0.5–5.1)	1.1 (0.4–4.2)
Ventilation (93/75)	141 (66–273)	138 (54–273)	1.5 (0.6–5.6)	1.6 (0.2–15.4)	1.3 (0.1–3.4)	1.3 (0.2–3.7)	1.1 (0.7–5.1)	1.1 (0.4–4.2)
Catecholamine (97/74)	146 (66–273)	135 (54–273)	1.5 (0.3–5.4)	1.6 (0.2–15.4)	1.3 (0.1–3.4)	1.2 (0.2–3.7)	1.1 (0.6–5.1)	1.1 (0.4–4.2)

**Table 3:** Spearman correlation coefficients and *p*-values for the correlation of plasma apoA-IV, triglyceride, cholesteryl ester (CE) and free cholesterol (FC) levels with biomarkers of inflammation.

Biomarker of Inflammation	ApoA-IV		TG		CE		FC	
	r	*p*-value	r	*p*-value	r	*p*-value	r	*p*-value
Leukocytes	−0.074	0.358	0.048	0.605	−0.137	0.136	0.099	0.284
Neutrophils	−0.060	0.459	0.065	0.491	−0.180	0.053	0.070	0.453
Basophils	0.070	0.392	0.147	0.113	−0.161	0.083	0.085	0.364
Eosinophils	0.034	0.674	0.152	0.102	−0.178	0.055	0.031	0.738
Monocytes	−0.007	0.927	0.075	0.421	−0.227	0.014	0.012	0.899
Lymphocytes	−0.035	0.669	0.224	0.015	−0.040	0.668	0.075	0.422
Immature granulocytes	−0.072	0.380	0.212	0.023	−0.143	0.127	0.122	0.195
Procalcitonin	0.094	0.250	0.120	0.198	−0.364	<0.001	0.174	0.061
C-reactive protein	−0.009	0.908	0.284	0.002	−0.043	0.639	0.229	0.012
IL-6	0.076	0.361	−0.065	0.487	−0.267	0.004	−0.080	0.391

## Data Availability

Data supporting reported results can be obtained from the corresponding author.

## References

[1] Olofsson S.O., Wiklund O., Boren J. (2007). Apolipoproteins A-I and B: Biosynthesis, role in the development of atherosclerosis and targets for intervention against cardiovascular disease. Vasc. Health Risk Manag..

[2] Florea G., Tudorache I.F., Fuior E.V., Ionita R., Dumitrescu M., Fenyo I.M., Bivol V.G., Gafencu A.V. (2022). Apolipoprotein A-II, a Player in Multiple Processes and Diseases. Biomedicines.

[3] Khovidhunkit W., Kim M.S., Memon R.A., Shigenaga J.K., Moser A.H., Feingold K.R., Grunfeld C. (2004). Effects of infection and inflammation on lipid and lipoprotein metabolism: Mechanisms and consequences to the host. J. Lipid Res..

[4] Kowalska K., Sabatowska Z., Forycka J., Mlynarska E., Franczyk B., Rysz J. (2022). The Influence of SARS-CoV-2 Infection on Lipid Metabolism-The Potential Use of Lipid-Lowering Agents in COVID-19 Management. Biomedicines.

[5] Zhao M., Luo Z., He H., Shen B., Liang J., Zhang J., Ye J., Xu Y., Wang Z., Ye D. (2021). Decreased Low-Density Lipoprotein Cholesterol Level Indicates Poor Prognosis of Severe and Critical COVID-19 Patients: A Retrospective, Single-Center Study. Front. Med..

[6] Barlage S., Gnewuch C., Liebisch G., Wolf Z., Audebert F.X., Gluck T., Frohlich D., Kramer B.K., Rothe G., Schmitz G. (2009). Changes in HDL-associated apolipoproteins relate to mortality in human sepsis and correlate to monocyte and platelet activation. Intensive Care Med..

[7] Hofmaenner D.A., Kleyman A., Press A., Bauer M., Singer M. (2022). The Many Roles of Cholesterol in Sepsis: A Review. Am. J. Respir. Crit. Care Med..

[8] Sharma N.K., Ferreira B.L., Tashima A.K., Brunialti M.K.C., Torquato R.J.S., Bafi A., Assuncao M., Azevedo L.C.P., Salomao R. (2019). Lipid metabolism impairment in patients with sepsis secondary to hospital acquired pneumonia, a proteomic analysis. Clin. Proteomics.

[9] Barker G., Leeuwenburgh C., Brusko T., Moldawer L., Reddy S.T., Guirgis F.W. (2021). Lipid and Lipoprotein Dysregulation in Sepsis: Clinical and Mechanistic Insights into Chronic Critical Illness. J. Clin. Med..

[10] Karakike E., Giamarellos-Bourboulis E.J., Kyprianou M., Fleischmann-Struzek C., Pletz M.W., Netea M.G., Reinhart K., Kyriazopoulou E. (2021). Coronavirus Disease 2019 as Cause of Viral Sepsis: A Systematic Review and Meta-Analysis. Crit. Care Med..

[11] Marques M.O., Abdo A., Silva P.B., Silva Junior A., Alves L.B.O., Costa J.V.G., Martin J., Bachour P., Baiocchi O.C.G. (2022). Soluble CD137 as a potential biomarker for severe COVID-19. Immunol. Lett..

[12] Begue F., Chemello K., Veeren B., Lortat-Jacob B., Tran-Dinh A., Zappella N., Snauwaert A., Robert T., Rondeau P., Lagrange-Xelot M. (2023). Plasma Apolipoprotein Concentrations Are Highly Altered in Severe Intensive Care Unit COVID-19 Patients: Preliminary Results from the LIPICOR Cohort Study. Int. J. Mol. Sci..

[13] Feingold K.R. (2023). The bidirectional interaction of COVID-19 infections and lipoproteins. Best Pract. Res. Clin. Endocrinol. Metab..

[14] Qu J., Ko C.W., Tso P., Bhargava A. (2019). Apolipoprotein A-IV: A Multifunctional Protein Involved in Protection against Atherosclerosis and Diabetes. Cells.

[15] Kohan A.B., Wang F., Lo C.M., Liu M., Tso P. (2015). ApoA-IV: Current and emerging roles in intestinal lipid metabolism, glucose homeostasis, and satiety. Am. J. Physiol. Gastrointest. Liver Physiol..

[16] Dallongeville J., Lebel P., Parra H.J., Luc G., Fruchart J.C. (1997). Postprandial lipaemia is associated with increased levels of apolipoprotein A-IV in the triacylglycerol-rich fraction and decreased levels in the denser plasma fractions. Br. J. Nutr..

[17] Seishima M., Noma A., Torizawa H., Muto Y. (1988). Changes of serum apolipoprotein levels after oral administration of fat in human subjects. Atherosclerosis.

[18] Recalde D., Ostos M.A., Badell E., Garcia-Otin A.L., Pidoux J., Castro G., Zakin M.M., Scott-Algara D. (2004). Human apolipoprotein A-IV reduces secretion of proinflammatory cytokines and atherosclerotic effects of a chronic infection mimicked by lipopolysaccharide. Arterioscler. Thromb. Vasc. Biol..

[19] Vowinkel T., Mori M., Krieglstein C.F., Russell J., Saijo F., Bharwani S., Turnage R.H., Davidson W.S., Tso P., Granger D.N. (2004). Apolipoprotein A-IV inhibits experimental colitis. J. Clin. Investig..

[20] Shearston K., Tan J.T.M., Cochran B.J., Rye K.A. (2022). Inhibition of Vascular Inflammation by Apolipoprotein A-IV. Front. Cardiovasc. Med..

[21] Roula D., Theiler A., Luschnig P., Sturm G.J., Tomazic P.V., Marsche G., Heinemann A., Sturm E.M. (2020). Apolipoprotein A-IV acts as an endogenous anti-inflammatory protein and is reduced in treatment-naive allergic patients and allergen-challenged mice. Allergy.

[22] Khovidhunkit W., Duchateau P.N., Medzihradszky K.F., Moser A.H., Naya-Vigne J., Shigenaga J.K., Kane J.P., Grunfeld C., Feingold K.R. (2004). Apolipoproteins A-IV and A-V are acute-phase proteins in mouse HDL. Atherosclerosis.

[23] Shi T., Bai J., Yang D., Huang L., Fan H.F., Zhang D.W., Liu T., Lu G. (2022). Identification of candidate biomarkers for severe adenovirus community-acquired pneumonia by proteomic approach. Heliyon.

[24] Flannery A.H., Li X., Delozier N.L., Toto R.D., Moe O.W., Yee J., Neyra J.A. (2021). Sepsis-Associated Acute Kidney Disease and Long-term Kidney Outcomes. Kidney Med..

[25] Kronenberg F. (2017). Apolipoprotein L1 and apolipoprotein A-IV and their association with kidney function. Curr. Opin. Lipidol..

[26] Lingenhel A., Lhotta K., Neyer U., Heid I.M., Rantner B., Kronenberg M.F., Konig P., von Eckardstein A., Schober M., Dieplinger H. (2006). Role of the kidney in the metabolism of apolipoprotein A-IV: Influence of the type of proteinuria. J. Lipid Res..

[27] Romanova Y., Laikov A., Markelova M., Khadiullina R., Makseev A., Hasanova M., Rizvanov A., Khaiboullina S., Salafutdinov I. (2020). Proteomic Analysis of Human Serum from Patients with Chronic Kidney Disease. Biomolecules.

[28] Cheng C.W., Chang C.C., Chen H.W., Lin C.Y., Chen J.S. (2018). Serum ApoA4 levels predicted the progression of renal impairment in T2DM. Eur. J. Clin. Investig..

[29] Singer M., Deutschman C.S., Seymour C.W., Shankar-Hari M., Annane D., Bauer M., Bellomo R., Bernard G.R., Chiche J.D., Coopersmith C.M. (2016). The Third International Consensus Definitions for Sepsis and Septic Shock (Sepsis-3). JAMA.

[30] Bone R.C. (1995). Sepsis, sepsis syndrome, and the systemic inflammatory response syndrome (SIRS). Gulliver in Laputa. JAMA.

[31] Bligh E.G., Dyer W.J. (1959). A rapid method of total lipid extraction and purification. Can. J. Biochem. Physiol..

[32] Peschel G., Grimm J., Muller M., Horing M., Krautbauer S., Weigand K., Liebisch G., Buechler C. (2022). Sex-specific changes in triglyceride profiles in liver cirrhosis and hepatitis C virus infection. Lipids Health Dis..

[33] Horing M., Ejsing C.S., Krautbauer S., Ertl V.M., Burkhardt R., Liebisch G. (2021). Accurate quantification of lipid species affected by isobaric overlap in Fourier-transform mass spectrometry. J. Lipid Res..

[34] Horing M., Ejsing C.S., Hermansson M., Liebisch G. (2019). Quantification of Cholesterol and Cholesteryl Ester by Direct Flow Injection High-Resolution Fourier Transform Mass Spectrometry Utilizing Species-Specific Response Factors. Anal. Chem..

[35] Hofmaenner D.A., Arina P., Kleyman A., Page Black L., Salomao R., Tanaka S., Guirgis F.W., Arulkumaran N., Singer M. (2023). Association Between Hypocholesterolemia and Mortality in Critically Ill Patients with Sepsis: A Systematic Review and Meta-Analysis. Crit. Care Explor..

[36] Feingold K.R., Grunfeld C., Feingold K.R., Anawalt B., Blackman M.R., Boyce A., Chrousos G., Corpas E., de Herder W.W., Dhatariya K., Dungan K., Hofland J. (2000). The Effect of Inflammation and Infection on Lipids and Lipoproteins. Endotext.

[37] Zhang X., Liu H., Hashimoto K., Yuan S., Zhang J. (2022). The gut-liver axis in sepsis: Interaction mechanisms and therapeutic potential. Crit. Care.

[38] Li X., Xu M., Liu M., Ji Y., Li Z. (2015). TNF-alpha and IL-6 inhibit apolipoprotein A-IV production induced by linoleic acid in human intestinal Caco2 cells. J. Inflamm..

[39] Ghenu M.I., Dragos D., Manea M.M., Ionescu D., Negreanu L. (2022). Pathophysiology of sepsis-induced cholestasis: A review. JGH Open.

[40] Sun J.K., Zhang Q., Shen X., Zhou J., Wang X., Zhou S.M., Mu X.W. (2022). Integrin alphaEbeta7 is involved in the intestinal barrier injury of sepsis. Aging.

[41] Orso E., Moehle C., Boettcher A., Szakszon K., Werner T., Langmann T., Liebisch G., Buechler C., Ritter M., Kronenberg F. (2007). The satiety factor apolipoprotein A-IV modulates intestinal epithelial permeability through its interaction with alpha-catenin: Implications for inflammatory bowel diseases. Horm. Metab. Res..

[42] Peignon G., Thenet S., Schreider C., Fouquet S., Ribeiro A., Dussaulx E., Chambaz J., Cardot P., Pincon-Raymond M., Le Beyec J. (2006). E-cadherin-dependent transcriptional control of apolipoprotein A-IV gene expression in intestinal epithelial cells: A role for the hepatic nuclear factor 4. J. Biol. Chem..

[43] Buechler C., Aslanidis C. (2020). Role of lipids in pathophysiology, diagnosis and therapy of hepatocellular carcinoma. Biochim. Biophys. Acta Mol. Cell Biol. Lipids.

[44] Wang P.W., Hung Y.C., Wu T.H., Chen M.H., Yeh C.T., Pan T.L. (2017). Proteome-based identification of apolipoprotein A-IV as an early diagnostic biomarker in liver fibrosis. Oncotarget.

[45] Seishima M., Nishimuraa M., Moriwakib H., Mutob Y., Nomaa A. (1995). Reduced intestinal apo A-IV mRNA levels in patients with liver cirrhosis. Int. Hepatol. Commun..

[46] Reisinger A.C., Schuller M., Sourij H., Stadler J.T., Hackl G., Eller P., Marsche G. (2021). Impact of Sepsis on High-Density Lipoprotein Metabolism. Front. Cell Dev. Biol..

[47] Taylor R., Zhang C., George D., Kotecha S., Abdelghaffar M., Forster T., Santos Rodrigues P.D., Reisinger A.C., White D., Hamilton F. (2024). Low circulatory levels of total cholesterol, HDL-C and LDL-C are associated with death of patients with sepsis and critical illness: Systematic review, meta-analysis, and perspective of observational studies. eBioMedicine.

[48] Massy Z.A., de Zeeuw D. (2013). LDL cholesterol in CKD—To treat or not to treat?. Kidney Int..

[49] Sam R., Zhang L., Tuot D.S., Chaudhry R. (2023). The Decrease in Serum Total Cholesterol and Low-Density Lipoprotein (LDL) Concentrations with the Initiation of Hemodialysis Despite a Concomitant Increase in Serum Albumin Concentrations. Cureus.

[50] Stasi A., Franzin R., Fiorentino M., Squiccimarro E., Castellano G., Gesualdo L. (2021). Multifaced Roles of HDL in Sepsis and SARS-CoV-2 Infection: Renal Implications. Int. J. Mol. Sci..

[51] Stangl S., Kollerits B., Lamina C., Meisinger C., Huth C., Stockl A., Dahnhardt D., Boger C.A., Kramer B.K., Peters A. (2015). Association between apolipoprotein A-IV concentrations and chronic kidney disease in two large population-based cohorts: Results from the KORA studies. J. Intern. Med..

[52] Holven K.B., Roeters van Lennep J. (2023). Sex differences in lipids: A life course approach. Atherosclerosis.

[53] Laclaustra M., Lopez-Garcia E., Civeira F., Garcia-Esquinas E., Graciani A., Guallar-Castillon P., Banegas J.R., Rodriguez-Artalejo F. (2018). LDL Cholesterol Rises with BMI Only in Lean Individuals: Cross-sectional U.S. and Spanish Representative Data. Diabetes Care.

[54] Hussain A., Ali I., Kaleem W.A., Yasmeen F. (2019). Correlation between Body Mass Index and Lipid Profile in patients with Type 2 Diabetes attending a tertiary care hospital in Peshawar. Pak. J. Med. Sci..

[55] Verges B., Guerci B., Durlach V., Galland-Jos C., Paul J.L., Lagrost L., Gambert P. (2001). Increased plasma apoA-IV level is a marker of abnormal postprandial lipemia: A study in normoponderal and obese subjects. J. Lipid Res..

[56] Sun Z., Larson I.A., Ordovas J.M., Barnard J.R., Schaefer E.J. (2000). Effects of age, gender, and lifestyle factors on plasma apolipoprotein A-IV concentrations. Atherosclerosis.

